# Supplementary material for: Antecedents of Gen Z’s green purchase intention in Vietnam’s fashion industry with the moderating role of greenwash perception
Source: PLoS One. 2025 May 30;20(5):e0324923. doi: 10.1371/journal.pone.0324923 (PMC12124538; doi:10.1371/journal.pone.0324923)
Supplement: Attachment — Submitted filename: Review PONE-D-24-05489.docx [file pone.0324923.s002.docx]

**Manuscript Number: PONE-D-24-05489**

**Title: Antecedents of gen z’s green purchase intention in Vietnam’s fashion industry with the moderating role of greenwash perception**

1. Introduction:

The authors have described the current situation in the Vietnamese context. However, green purchase intention is not a new topic. The authors have failed to examine the literature and the research gap has been poorly emphasized in the introduction. One paragraph to include the academic/ theoretical background and explain why authors want to focus on this topic is necessary. Furthermore, authors might consider including the research objectives at the end of the introduction.

2. Literature review

Again, since green purchase intention and TPB have been excessively examined, authors should focus more on how to apply the model in the fashion industry for GenZ consumers. What has been explained in the literature review is too general. Furthermore, it is better to rewrite the part of the moderating hypothesis. This hypothesis development part seems to explain the direct effects only.

3. Methodology

Authors should include a few sentences to describe how they approach the participants to collect data.

93% is not the response rate. It is the completion rate.

4. Conclusions and implications

This section should be divided into 3 parts: conclusion, practical implications, and limitations and future research. The conclusion part should discuss whether the research objectives have been completed or not and mention the contribution of this study.

The authors have written quite lengthy paragraphs for the implications. However, these implications are too general. Please rewrite the parts.

5. Language and formatting

The language quality is not at the academic level. there are many grammar mistakes. Please consider using a proofreading service.
