# Supplementary material for: Antecedents of Gen Z’s green purchase intention in Vietnam’s fashion industry with the moderating role of greenwash perception
Source: PLoS One. 2025 May 30;20(5):e0324923. doi: 10.1371/journal.pone.0324923 (PMC12124538; doi:10.1371/journal.pone.0324923)
Supplement: Attachment — Submitted filename: Detailed response to reviewers.docx [file pone.0324923.s004.docx]

**RESPONSE TO REVIEWER**

**Title: Antecedents of gen z’s green purchase intention in Vietnam’s fashion industry with the moderating role of greenwash perception**

1. Introduction:

The authors have described the current situation in the Vietnamese context. However, green purchase intention is not a new topic. The authors have failed to examine the literature and the research gap has been poorly emphasized in the introduction. One paragraph to include the academic/ theoretical background and explain why authors want to focus on this topic is necessary. Furthermore, authors might consider including the research objectives at the end of the introduction.

**Author response:** Thank you for the feedback. We understand that the theoretical background and research gap needs to be emphasized to highlight that the study is necessary, and the objectives of the study should be done. In response to the feedback, we have added a paragraph outline the theoretical background and the important of this topic in the manuscript. This adjustment will contribute to a more comprehensive introduction, creating the conditions for a more informative reading experience.

**Change in manuscript:**

1. Introduction

The majority of studies investigating green purchasing intention (GPI) have adopted a general approach, with limited research specifically focusing on the fashion industry (Casalegno et al., 2022; Yadav & Pathak, 2016), or conducted in developed countries such as China (Lu et al., 2022) and Germany (Rausch & Kopplin, 2021). To address these gaps, this study delves into GPI within the fashion industry in Vietnam, a developing country. To address these gaps, this study delves into green purchase intention within the fashion industry in Vietnam, a developing nation. Furthermore, previous studies have proposed different models of Greenwashing (Szabo & Webster, 2020; Nguyen et al., 2019), often treating Greenwash Perception as an independent variable. However, calls have been made to investigate the moderating role of Greenwash Perception to see the relationship between the variables in the extended TPB and Green Purchase Intention, this study will close a gap in previous research by considering Greenwash Perception as a moderator. Recognizing the limitations of prior research, our study focuses on the influence of greenwash on the green purchase intention of consumers, particularly Gen Z, a key target group for fashion brands.

This study examined the impact of 4 factors (Attitudes, Subjective Norms, Perceived Behavioral Control, and Perceived Consumer Effectiveness) on the Green Purchase Intention of Gen Z when the greenwashing perception is a moderating variable, applying the TPB and analyzing data through the PLS-SEM method. The following section of the study will include the literature review and hypotheses development in Section 2. The methodology will be explained in Section 3, and after that, the data analysis techniques and results will be shown in Section 4. The last part provided the implications, conclusions, and study limitations.

To achieve the research objectives, the research will answer the following questions:

(1) What factors influence Gen Z's intention to consume green fashion products in Vietnam? What is the level of impact of these factors on Gen Z's intention to consume green fashion in Vietnam?

(2) Does the perception of “greenwashing” moderate the relationship between factors and green fashion consumption intention? What is the direction and level of moderating of "greenwashing"?

2. Literature review:

Again, since green purchase intention and TPB have been excessively examined, authors should focus more on how to apply the model in the fashion industry for GenZ consumers. What has been explained in the literature review is too general. Furthermore, it is better to rewrite the part of the moderating hypothesis. This hypothesis development part seems to explain the direct effects only.

**Author response:** Thank you for the comment and suggestion. It is true that the Theory of Planned Behavior (TPB) has been widely discussed in various studies. Therefore, we have added more details on how previous studies have applied this model to research on fashion for Gen Z consumers. Additionally, we have focused more on explaining the moderating role of Greenwash Perception to enhance the understanding of readers and ensure consistency throughout the study.

**Change in manuscript:**

**2. Literature review and hypothesis development**

In the context of fashion and sustainable fashion, TPB has proven to be a useful model for forecasting consumer intentions, particularly Gen Z consumers. Phau et al. (2015) used TPB analysis to assess the reasons behind purchasing luxury fashion clothes manufactured in sweatshops. They looked at two scenarios: the desire to pay more for fashion apparel that is not sweatshop-made and the desire to avoid purchasing luxury brands that are. Rausch et al. (2020) endeavored to verify TPB in the context of green product consumption by utilizing the expanded version of TPB, which encompasses environmental concerns. The authors discovered that while customers' concerns about greenwashing have a detrimental impact on this relationship, attitude toward sustainable clothes influences purchase intention most. A model based on the extension of TPB was developed in Ge's (2024) research to determine the elements of influencer marketing that impact the primary reasons why Gen Z consumers intend to buy sustainable fashion products. These factors include attitudes toward sustainable fashion influencers, friends' opinions influenced by influencers, and the affordable products that influencers recommend.

***2.5 Moderating role of Greenwash Perception***

Green marketing has developed in response to consumers' increased desire for products and services that have a positive environmental impact as they become more concerned about environmental protection in the context of globalization (Mishra & Sharma, 2010). Using green marketing methods has become standard practice for companies looking to get a competitive edge and draw in eco-aware customers. It is imperative to acknowledge that not all assertions made via green marketing precisely mirror an organization's true environmental practices, which may result in "greenwashing" (Szabo & Webster, 2021). The phrase "greenwashing" refers to the false and dishonest representations made by certain companies regarding the environmental friendliness of their products or services (Parguel et al., 2011). It is important to recognize greenwashing because, in the absence of it, well-meaning consumers may be duped into believing that the choices they make would contribute to environmental preservation. The term "greenwashing" actually has a lot of definitions, especially in the last several years. Certain studies have resulted in advancements and improved precision in comprehending the notion of greenwashing. Walker et al. (2012) define greenwashing as the distinction made by a business between "substantial" and "symbolic" social actions; the former narrows the focus from environmental issues to the latter through the use of image advertising, confusing terminology, and imagery.

While greenwashing can theoretically help businesses boost earnings and improve their standing and reputation, it has a detrimental effect on the environment and sustainable social behaviors. Numerous studies have shown the detrimental effects of greenwashing, as the trust that stakeholders have in green brands has been significantly damaged by corporations' attempts to utilize greenwashing in product promotion (Guo et al., 2017). In addition to having a detrimental effect on customer behavioral intentions, greenwashing also has a negative influence on business social responsibility and reputation (Cordero et al., 2021).

Recent years have seen a significant development in the reality of "greenwashing" in the fashion business, particularly as people's awareness of environmental preservation and sustainable living has grown. Studies have been done to look at the reasons behind greenwashing and how it impacts businesses, investors, and other stakeholders. Customers' capacity to distinguish between green advertising messaging and a company's true environmental commitment is known as their perception of greenwashing (Nyilasy, 2014). Put differently, customers' psychological assessments regarding a company's potential to misrepresent its environmental impact and hide its true environmental message are known as perceptions of greenwashing.

Limited research exists on how corporate greenwashing, within the TPB framework, influences consumer green purchase intentions. This study proposes a theoretical model where Greenwash Perception moderates the relationships between the core extended TPB constructs and Green Purchase Intention.

Consumers with positive attitudes towards green products are more likely to exhibit green purchase behavior (Suki, 2013). However, greenwash perception can moderate this relationship. If consumers perceive greenwashing, their positive attitude towards green products may weaken, leading to a decreased likelihood of green purchase.

Subjective norms represent societal influences on consumer behavior (De Pelsmacker et al., 2006). Consumers are affected by their social circles and the perceived social acceptability of various activities. Greenwash perception might help to attenuate this association. If consumers learn of a company's greenwashing, they may be less likely to buy the product because of fear of social repercussions for supporting ecologically hazardous actions.

Perceived behavioral control relates to customers' belief in their capacity to do a given activity (De Pelsmacker, 2006). In the context of green purchase intention, this translates to a consumer's confidence in their ability to make green choices. Studies have shown a positive correlation, indicating that consumers who feel empowered to make a difference through their purchases are more likely to do so. However, when consumers encounter greenwashing tactics, it can erode their trust in companies' environmental claims. This skepticism can lead them to question the effectiveness of their individual choices, hence, discouraging consumers from engaging in green purchasing despite their perceived ability to do so.

Similar to Perceived Behavioral Control, Perceived Consumer Effectiveness plays a role in influencing purchasing intention, refers to a consumer's belief in the impact of their actions on achieving a desired outcome. In the context of green practices, this translates to a consumer's belief that their green purchases can make a difference for the environment. However, Greenwash Perception may undermine this relationship. Exposed to greenwashing, consumers may doubt the true environmental benefit of green products, leading them to question the effectiveness of their choices despite a strong belief in making a difference.

3. Methodology:

Authors should include a few sentences to describe how they approach the participants to collect data.

93% is not the response rate. It is the completion rate.

**Author response**: Thank you for the comment. We have reviewed the manuscript and made the necessary adjustments.

**Change in manuscript:**

**3.4 Participants**

The authors collected data through an online survey using Google Forms and distributed it on social media platforms. Out of the 500 responses that were recorded, 467 proved to be valid after removing the invalid ones. With a 93% completion rate, this shows a high level of participant engagement. These comprise 28.9% male and 71.1% female. Of the Gen Z participants surveyed, 76.9% are 19 - 22 years old, 17.3% are between the ages of 15 and 18, and 5.8% are between the ages of 23 and 26. The descriptive statistics of the survey are shown in the table 1 below.

4. Conclusions and implications:

This section should be divided into 3 parts: conclusion, practical implications, and limitations and future research. The conclusion part should discuss whether the research objectives have been completed or not and mention the contribution of this study.

The authors have written quite lengthy paragraphs for the implications. However, these implications are too general. Please rewrite the parts.

**Author response**: Thank you for the comment and suggestions. Based on the comments, we have divided into 3 parts: Conclusions, implications, and limitations and future research direction. Moreover, we also made some adjustments to the implications part to make it more specific and focused.

**Change in manuscript:**

**5. Conclusions and Implications**

**5.1. Conclusions**

This study proposes an extensive model to show how Gen Z customers in Vietnam's fashion sector intend to make green purchases. According to the study's model, the antecedents of intention to buy green products include attitude, subjective norms, perceived behavior control, and perceived consumer effectiveness. Furthermore, by adding greenwash perception as a moderator variable, the study unveils the nuanced influence of greenwash perception as a moderator, significantly impacting the relationships between key determinants such as attitudes and perceived consumer effectiveness. The negative influence of greenwash perception shows an increase in the awareness of Generation Z about the sustainability of products but also sensitivity to insincere green promises.

The study's findings reveal a robust relationship between various factors and the purchase intentions of young consumers, particularly in the context of green products, with a specific focus on green clothing. The predictive elements encompass attitudes and subjective norms, perceived behavioral control. The predictive elements encompass Attitudes and Subjective Norms, Perceived Behavioral Control, and Perceived Consumer Effectiveness. This outcome is consistent with existing research across diverse domains, including studies on green vegetable purchasing in developing countries (Nguyen et al., 2019), the acquisition of organic food by young consumers aged 18-30 in China (Ahmed et al., 2021), and the purchase intentions of Chinese consumers towards green products (Liu et al., 2020).

The alignment of our results with these varied research efforts underscores the applicability and relevance of the theory of planned behavior to our study. The empirical evidence supports the contention that attitudes, subjective norms, and perceived behavioral control exert positive effects on the purchase intentions of young consumers, reinforcing the suitability of the chosen theoretical framework and its associated measures for our investigation.

Some studies assert a direct influence of greenwashing on the intention to make environmentally friendly purchases, as evidenced by Zhang et al. (2018). Simultaneously, there is a body of research exploring the role of greenwashing as a moderator (Zaidi et al., 2019; Bulut et al., 2021; Wu & Liu, 2022). This present study delves into the moderating function of perceived greenwash. In our ongoing study, we are specifically exploring the moderating role of perceived greenwash. Greenwashing perceptions indeed appear to influence consumer decisions on the intention-formation level as they were found to moderate the relation between attitude and green purchase intention according to Rausch & Kopplin (2021), towards sustainable clothes and purchase intention for sustainable clothes, the same result is also proved in our study. Moreover, Rausch & Kopplin's study (2021) yielded similar results to our investigation, suggesting insufficient evidence to demonstrate that perceived greenwash moderates the relationship between subjective norms and green purchase intentions. A research by Rejikumar (2016) has also demonstrated the same negative moderator role of Perceived Greenwash between Perceived Consumer Effectiveness and Green Purchase Intention.

5.2. Theoretical implications

The research has developed a model to study the factors influencing green purchase intention, with the addition of the moderating effect of greenwashing perception on these relationships with a focus on Generation Z in Vietnam's fashion sector. This contributes to the theoretical diversity of topics related to green consumer behavior and enriches the knowledge of consumer psychology in the market.

Furthermore, there remains a limited number of studies investigating the moderating effect of greenwash perception on various relationships. Our research has contributed to this field by focusing on a narrower scope, specifically targeting Generation Z individuals in Vietnam within the fashion industry. By doing so, we have provided unique insights into a demographic and market that have been underexplored in existing literature.

5.3. Managerial implications

The implications drawn from the research have significant practical relevance for various stakeholders in the context of Greenwashing among Generation Z in Vietnam's fashion industry.

Firstly, there is a rising trend among Gen Z to prioritize sustainable and eco-friendly choices. The research substantiates this by revealing that a positive influence on green purchase intention is derived from factors such as attitude, subjective norms, perceived behavioral control, and perceived consumer effectiveness. Consequently, understanding Gen Z's inclination toward green purchasing behavior and environmental concerns becomes pivotal for businesses in Vietnam's fashion industry. By comprehending the factors influencing green purchase intention and the moderating role of Greenwash Perception, companies can tailor their marketing, communication strategies, and product offerings to better resonate with the environmentally conscious Gen Z consumers. Businesses that align with these values can gain a competitive advantage in the market, empowering them to build brand loyalty, enhance corporate responsibility, and contribute to the overall sustainability goals of the fashion industry in Vietnam.

Secondly, the study underscores the importance for companies to adopt genuine and substantial sustainability measures, avoiding the pitfalls of greenwashing. The findings suggest that consumers' perception of greenwashing negatively impacts their willingness to purchase related products. To mitigate this risk, companies are advised to embrace authenticity in their communication and marketing strategy, steering clear of exaggeration or concealment. The study emphasizes that exposure to greenwashing can lead to severe repercussions on a brand's reputation and the wider consumer market, urging companies to carefully weigh the risks associated with such deceptive practices.

This highlights an opportunity for businesses and the fashion industry in Vietnam to reevaluate and enhance their green communication and practices, aiming to bolster trust and commitment from Generation Z. Furthermore, the research underscores the imperative of raising awareness and educating consumers about Greenwashing, enabling them to discern between authentic environmental commitments and deceptive green communication strategies. This not only contributes to cultivating a more sustainable market but also stimulates innovation and creativity within Vietnam's fashion industry.

6. Limitations and future reseach direction

Although this study provides important new information about how Vietnam's Generation Z is affected by Greenwashing, it also highlights its shortcomings and makes recommendations for future research areas. Some restrictions could be taken into account for more studies. The awareness and consumption behaviors of customers vary depending on the industry and the nation. Consequently, to obtain a more multifaceted understanding of the alterations in consumer behavior, future research might broaden the scope of studies in other sectors and in other nations. In the conceptual model, we just use a limited number of variables based on the extended TPB framework, therefore, future research can use other factors that influence consumers' green consumption intention. Furthermore, the focus on the fashion industry and Generation Z in Vietnam implies that future studies should broaden their scope for a more holistic understanding of this issue to add depth and dimension to the topic, contributing significantly to a more comprehensive understanding of the influence of Greenwashing on the shopping behavior of Generation Z in Vietnam.

5. Language and formatting

The language quality is not at the academic level. There are many grammar mistakes. Please consider using a proofreading service.

**Author response**: Thank you for the insightful comment. We have carefully checked the grammar mistakes and rewrite sentences.
