# Supplementary material for: Antecedents of Gen Z’s green purchase intention in Vietnam’s fashion industry with the moderating role of greenwash perception
Source: PLoS One. 2025 May 30;20(5):e0324923. doi: 10.1371/journal.pone.0324923 (PMC12124538; doi:10.1371/journal.pone.0324923)
Supplement: Attachment — Submitted filename: Response to Reviewers (R2).docx [file pone.0324923.s005.docx]

**RESPONSE TO REVIEWER**

**Title: Antecedents of gen z’s green purchase intention in Vietnam’s fashion industry with the moderating role of greenwash perception**

**Reviewer #3:**

1. The abstract showed a relevant study in the paper, clear objective, key findings and results. There are some following points that should rewrite for improvement:

- The methodology should describe more information such as number of respondents, how to collect data, how to process the collected data,...

**Authors’ response:** Thank you for your feedback on the methodology section. We have now included details on the number of respondents, data collection process, and data analysis methods to ensure clarity.

**Change in manuscript:**

**Abstract**

While buying sustainable fashion items is becoming more and more popular worldwide, the concept of green fashion is still relatively new in Vietnam. Besides, many fashion brands use “greenwashing” to deceive consumers and encourage their purchases. This study aims to examine the drivers of green purchasing intention among Gen Z in Vietnam’s fashion industry with greenwash perception as the moderating factor. The conceptual model was analyzed using structural equation modeling with the bootstrapping method based on data gathered from 467 Vietnamese Gen Z customers.

- The implications should be clearly.

**Authors’ response:** Thank you for your recommendation, the authors have improved the abstract as follows:

**Change in manuscript:**

**Abstract**

The findings reveal significant positive influences of attitude, subjective norms, perceived behavioral control, and perceived consumer effectiveness on green purchase intention. Greenwash perception serves as a significant negative moderator, enhancing the relationship between the determinants and green purchasing intention. The results hold significant implications for businesses, encouraging them to embrace transparent and genuine sustainability practices instead of engaging in greenwashing. This can be achieved by clearly communicating their initiatives, offering third-party certifications, and conducting educational campaigns. The study provides original contributions to the existing body of literature and offers recommendations for future research in the context of developing countries.

2. The introduction presented the background of the study, clearly gaps, theories and research questions. The introduction should consider the citations and explain how to apply TBP and PLS-SEM in this study.

**Authors’ response:** Thank you for your valuable feedback on the introduction. We have carefully reviewed the comment and made the following improvements. First, ưe have included relevant citations to strengthen the background and theoretical foundation of the study. Specifically, we have cited recent key studies that highlight the gaps in the existing literature and justify the need for this research. Second, we understand that it is important to explain the role of TPB and the use of PLS SEM as our primary analytical method, so that we have made several changes to our introductory.

**Change in manuscript:**

**Introduction**

Green consumption has become a global trend, originating in developed countries and gradually spreading to developing nations like Vietnam (Thi Tuyet Mai, 2019). To promote environmental conservation, the United Nations introduced the Sustainable Development Goals (SDGs), with Goal 12 emphasizing sustainable production and consumption to reduce the ecological footprint. While researchers have extensively studied green consumption in various fields (Bryła, 2019; Ahmed et al., 2020), there is still limited research on sustainable fashion consumption, particularly in Asia's emerging markets. Consequently, to broaden the body of current research, this study focuses on the Vietnamese fashion sector, where the idea of "green fashion" was still relatively new.

In Vietnam, the government has implemented the National Strategy for Green Growth for 2021–2030 with a vision for 2050. The strategy includes initiatives to encourage sustainable consumption and promote greener lifestyles. Alongside rapid economic growth and a booming fashion sector (Nayak, 2021), Vietnamese consumers are increasingly aware of sustainability. However, the sustainable fashion industry in Vietnam faces significant challenges, including high production costs, limited resources (Zhang et al., 2018; Jackman & Moore, 2021), and deceptive practices like greenwashing, where companies create false impressions of environmental friendliness (Nguyen et al., 2019). Additionally, Vietnam still lacks comprehensive regulations and enforcement mechanisms to address these issues effectively. To be more precise, greenwashing refers to creating a false impression or presenting misleading information about a company's products being environmentally friendly. It arises from combining poor environmental performance with positive communication about sustainability (Nguyen et al., 2019). While greenwashing has been widely studied, there is a notable lack of research on its prevalence in the fashion industry within developing Asian countries (Yang et al., 2020). This is crucial, as consumers are often willing to pay more for products from companies genuinely committed to sustainability (Chen et al., 2018).

The research focuses on Generation Z (born between 1997 and 2012) for their strong awareness of ethical and environmental issues. This generation is often willing to pay more for eco-friendly products and has a significant influence on market trends (Casalegno et al., 2022; Anh Do et al., 2023). However, despite their environmentally conscious attitudes, Gen Z remains the largest consumer group in the fast fashion industry, accounting for about 40% of global sales. By 2025, Gen Z will include 2 billion individuals worldwide and will make up approximately 25% of Vietnam’s labor force, significantly influencing household and market consumption decisions (Do et al., 2023). Given their dual role as eco-conscious consumers and primary contributors to fast fashion consumption, it is critical to examine Gen Z's green purchase intentions in Vietnam’s fashion sector.

Therefore, most studies on green purchasing intention have taken a general approach, with limited focus on the fashion industry (Casalegno et al., 2022; Yadav & Pathak, 2016) or specific populations like Gen Z. Furthermore, while greenwashing has been studied as an independent variable (Nguyen et al., 2019; Szabo & Webster, 2020), there is a lack of research exploring its moderating role in shaping consumer behavior. To address these gaps, this study focuses on the Vietnamese fashion industry and investigates the influence of greenwashing perception on Gen Z's intention to purchase green fashion products.

To achieve the research concern, the research will answer the following questions:

***RQ1:*** How do four drivers (attitudes, subjective norms, perceived behavioral control, and perceived consumer effectiveness) influence the Gen Z's green purchase intention in Vietnam’s fashion industry?

***RQ2***: How does the perception of greenwashing moderate the relationship between these factors and Gen Z’s intention to consume green fashion products?

This study provides both theoretical and practical contributions by addressing these questions. By extending the Theory of Planned Behavior (TPB) model with four drivers (attitudes, subjective norms, perceived behavioral control, and perceived consumer effectiveness) and underscoring the role of moderating variable of greenwashing perception serving, this study provides a thorough understanding of factors influencing green purchasing intention among Gen Z in Vietnam’s fashion industry. The research was utilized the extension of TPB and used the PLS-SEM method for data analysis. The following section of the study will include literature review and the development of hypotheses. The methodology will be explained in Section 3, and after that, the data analysis techniques and results will be shown in Section 4. The last part provided the conclusions, implications, and study limitations.

3. The literature review showed the TPB and developed 8 hypotheses in which greenwash perception is the moderators.

- The review should streamline the structure with subheading consisting the concepts, theories, previous research and hypotheses development.

**Authors’ response:** Thank you for your constructive feedback. We have reorganized the literature review to include clear subheadings involve: (1) Greenwashing and Greenwash Perception, (2) Green Purchase Intention, (3) Extended Theory of Planned Behavior, and (4) Hypothesis Development. This restructuring ensures a logical and coherent presentation of the key elements.

- The term of greenwash perception, purchase intention should mention in the concept and more clearly. The recent research should argument more by evidence citations.

**Authors’ response:** Thank you for your constructive feedback. The authors have also expanded the definitions and discussions of greenwash greenwash perception and green purchase intention. These terms are clearly explained under each subheading, highlighting their importance to the study.

**Changes in manuscript:**

**2. Literature Review and Hypothesis Development**

***2.1. Greenwashing and Greenwashing Perception***

Green marketing has developed in response to consumers' increased desire for products and services that have a positive environmental impact as they become more concerned about environmental protection in the context of globalization (Szabo & Webster, 2020). Using green marketing methods has become standard practice for companies looking to get a competitive edge and draw in eco-aware customers. It is imperative to acknowledge that not all assertions made via green marketing precisely mirror an organization's true environmental practices, which may result in "greenwashing" (Szabo & Webster, 2021). The phrase "greenwashing" refers to the false and dishonest representations made by certain companies regarding the environmental friendliness of their products or services (Parguel et al., 2011). It is important to recognize greenwashing because, in the absence of it, well-meaning consumers may be duped into believing that their choices would contribute to environmental preservation. The term "greenwashing" has a lot of definitions, especially in the last several years. The practice of misleadingly disclosing "green" activities is known as "greenwashing" (Lee & Raschke, 2023; Seele & Schultz, 2022). It may be dependent on external factors, incentives, or pressures that define the institutional context in which it takes place (Zharfpeykan, 2021; Velte, 2022; Seele & Schultz, 2022; Li et al., 2022). Recent years have seen a significant development in the reality of "greenwashing" in the fashion business, particularly as people's awareness of environmental preservation and sustainable living has grown (Munir & Mohan, 2022; Alizadeh et al., 2024).

A negative perception component, greenwash perception is defined as the public or consumers recognizing and perceiving inaccurate or deceptive comments made by businesses about their environmental actions (Chen & Chang, 2012). This view reflects people's misgivings about whether a corporation is carrying out its environmental protection obligations and their cynicism about the sincerity of the company's green marketing initiatives (Pomering & Johnson, 2009). Customers generally hold the view that environmentally friendly products are safer and more ecologically friendly (Pekersen & Canöz, 2022), which results in a generally favorable perception of these items (Park & Lin, 2020; Kement et al., 2023). Customers are more inclined to doubt advertising claims when they see examples of greenwashing, though, and this can have a detrimental effect on how they feel about the products (Chang & Hung, 2023). Thus, greenwash perception has emerged as a key metric for assessing consumer trust and business social responsibility initiatives in the present climate of growing environmental consciousness (Munir & Mohan, 2022).

***2.2. Green Purchase Intention***

Green purchase intention refers to the consumer’s willingness or readiness to purchase environmentally friendly products (Ruangkanjanases et al., 2020). When evaluating customers' green intentions, several factors are crucial, including beliefs, needs, values, motivation, knowledge, demography, and attitudes (Choi & Johnson, 2019). A person's inclination and desire to favor environmentally friendly products over non-eco-friendly ones is theorized while making a purchase decision (Spielmann, 2020). Customers identify their need for a product during the evaluation process, which influences their choice to buy (Sullivan and Kim, 2018). In the context of green purchasing, this intention manifests when consumers consciously decide to buy eco-friendly products, aligning their actions with a commitment to reducing environmental harm (Ahmed et al., 2022). Based on the TPB model, green purchase intention builds on the broader concept of general purchase intention by raising consumer awareness of environmental sustainability, health, and nature while concentrating on eco-friendly products (Ali et al., 2022). Research suggests that a positive attitude toward green products significantly enhances the likelihood of forming green purchase intention (Amoako et al., 2020). Green purchase intention varies across cultures, individuals, and genders, highlighting the contextual nature of consumer behavior (Sreen et al. 2018). Identifying and appreciating green attributes in products not only motivates consumers to make environmentally conscious decisions but also positions them as responsible citizens who contribute to broader societal changes (Hamzah & Tanwir, 2021; Wang, Zaman and Alvi, 2022).

4. The methodology showed the research measures, data collection, data analysis, participants. - The methodology should explain more how to build the scales and data processing.

**Authors’ response:** Thank you for your insightful feedback. Thank you for your insightful feedback. To enhance clarity, we have restructured the methodology section into three subheadings: (1) Data Collection and Sample, (2) Research Measures, and (3) Data Analysis*.* We have also added details on how the research scales were developed, including the process of adapting and validating items from previous studies in the Research Measure section. This section now also describes the rationale for selecting specific measurement items and their relevance to the study objectives.

**Changes in manuscript:**

**3. Methodology**

***3.1. Data collection and sample***

The authors collected data through a quota convenience sampling method. The survey targeted in this study were Vietnamese Generation Z customers, who were born between 1997 and 2012. Data collection was conducted anonymously through an online survey distributed via Google Forms across various media channels and forums. This approach helped maintain participant neutrality and enhance data accuracy. A total of 500 responses were recorded, of which 467 were deemed valid after excluding incomplete or inconsistent entries. The survey achieved a high completion rate of 93%, reflecting strong participant engagement.

Out of 467 appropriate respondents, there are 28.9% of male respondents compared to 71.1% female respondents. Of the Gen Z participants surveyed, 76.9% are 18 - 22 years old, 17.3% are between the ages of 13 and 17, and 5.8% are between the ages of 23 and 28. Most survey respondents reside in Northern Vietnam, with 343 individuals (73.4%), followed by Southern Vietnam with 66 respondents (14.1%), and Central Vietnam accounting for 9.4%. Regarding occupation, the largest group comprises students, totaling 434 participants (92.9%), followed by employees with 23 individuals (4.9%). Business owners, freelancers, and others make up 0.9%, 0.4%, and 0.9%, respectively. Most participants spend less than 500,000 VND per month on green products (84.2%), while a smaller proportion spend between 500,000 and 1,000,000 VND (14.1%), and only a very small percentage spends over 1,000,000 VND (1.7%). The descriptive statistics of the survey are shown in Table 1 below.

***3.2.*** ***Research Measures***

Multiple items on a 5-point Likert-type Scale (1 = "Strongly disagree" to 5 = "Strongly agree") will be used to measure each concept. Every item featured a distinct keyword that embodied the specific concept. The items were grounded in previously validated scales and existing research, clearly indicating the concept they were measuring.

Participants' attitudes toward green purchasing intentions use Paul & Modi (2016) and Kamalanon (2022) measures, as these scales are comprehensive and have been validated in prior research on environmental behavior. Subjective norms were measured using scales from Paul & Modi (2016), which are widely recognized for their reliability in capturing social influences on behavior. Items related to perceived behavioral control were taken from Paul & Modi's (2016) study, as their measures effectively address individuals' confidence in performing green behaviors. Measures for perceived consumer effectiveness originate from a scale developed by Yoo et al. (2013); Kang et al. (2013), chosen for their robust ability to gauge consumers' belief in their impact on environmental outcomes. For evaluating green purchasing intention, the authors use items established by Paul & Modi (2016). Finally, greenwashing perception was principally examined using measures developed by Chen & Chang (2012) and Rausch & Kopplin (2020), selected for their strong theoretical underpinnings and proven effectiveness in analyzing skepticism toward corporate environmental claims. To confirm the validity and clarity of the assessment measures, the authors first conducted a pilot testing phase that was utilized to be carried out with experienced participants before the formal data collection with 20 Vietnamese Gen Z customers. Minor changes were made to the questionnaire based on the comments received. This pretest will examine the completeness, clarity, language, structure, and appropriateness of the assessment items.

***3.3. Data analysis***

The proposed research model includes four variables from the TPB extended model and the moderated effect of perceptions of greenwashing, that affect consumers' intentions to make green purchases. The authors utilized the Partial Least Squares Structural Equation Modeling (PLS-SEM) method since it requires latent components and various indicators to quantify perception (Tran & Huang, 2022). PLS-SEM, which is based on the theory of structural equation modeling, has the following benefits: (1) PLS-SEM employs two independent models, the measurement model and the structural model, to effectively combine latent and observable variables into a single analytical framework (Inkpen & Birkenshaw, 1994). (2) It allows for causal-predictive analysis of complex data with minimum theoretical backing, avoiding the necessity for precise data distribution assumptions (Hair et al., 2019). (3) PLS-SEM shows better resistance to variation problems brought on by measurement mistakes than regression analysis, producing more accurate and dependable results (Hill et al., 2021). The collected data was processed following the procedures outlined by Hair et al. (2019), which included assessing the measurement model to ensure reliability and validity, evaluating the structural model to analyze relationships between constructs, and examining the moderating effects of greenwashing perception to understand its influence on green purchasing intentions. Lastly, the authors also used 5000 bootstrapped samples to investigate the modẻating effects within the proposed model.

- The content of participants should mention in research results.

**Authors’ response:** Thank you for your insightful feedback. We have refined the presentation of participant information. While general details about the participants remain in the methodology section, their specific characteristics and demographics are now included in the research results section to align with best practices.

**Changes in manuscript:**

**3. Methodology**

***3.1*** ***Research Measures***

Out of 467 appropriate respondents, there are 28.9% of male respondents compared to 71.1% female respondents. Of the Gen Z participants surveyed, 76.9% are 18 - 22 years old, 17.3% are between the ages of 13 and 17, and 5.8% are between the ages of 23 and 28. Most survey respondents reside in Northern Vietnam, with 343 individuals (73.4%), followed by Southern Vietnam with 66 respondents (14.1%), and Central Vietnam accounting for 9.4%. Regarding occupation, the largest group comprises students, totaling 434 participants (92.9%), followed by employees with 23 individuals (4.9%). Business owners, freelancers, and others make up 0.9%, 0.4%, and 0.9%, respectively. Most participants spend less than 500,000 VND per month on green products (84.2%), while a smaller proportion spend between 500,000 and 1,000,000 VND (14.1%), and only a very small percentage spends over 1,000,000 VND (1.7%). The descriptive statistics of the survey are shown in Table 1 below.

5. The results showed concisely reliability and value.

- The results should arrange the structures of subheading that are suitable for data processing.

**Authors’ response:** Thank you for your valuable suggestions regarding the results section. The results section has been reorganized with subheadings that align with the data processing steps, as follows: Hypothesis testing includes measurement model (Reliability, Validity Analysis), structural model, and moderating effects testing. The revised order reflects the standard practice in PLS-SEM analysis as it firstly check for the reliability and validity to ensure that the constructs and indicators are statistically sound. Then, testing the structural model to analyze relationships and determine whether hypotheses are supported.

- The results should explain the finding of the study.

**Authors’ response:** Thank you for your valuable feedback. We have expanded the Results section to provide a more detailed interpretation of the findings, ensuring that the key insights are clearly explained.

**Changes in manuscript:**

**4. Result**

***4.1.2 Structural model***

This article utilized PLS bootstrapping with 5000 bootstraps and 467 cases to enlighten the path coefficients and their significance. The PLS structural model and hypotheses are assessed by examining the significance of the path coefficients and the variance accounted for by the antecedent constructs.

Inner VIF was considered to ensure multicollinearity and data-related deviations. Diamantopoulos & Siguaw (2006) selected a multicollinearity tolerance level of 30% as the cut-off criterion based on guidelines suggesting tolerance values greater than 35% may cause multicollinearity problems. Based on the tolerance levels reported by Diamantopoulos & Siguaw (2006), Petter et al. (2007) recommend a 33% tolerance level, translating to a VIF of ≤ 3.3. Table 4 below demonstrates the inner VIF, in which all values are smaller than 3.3. Hence there is no issue of multicollinearity.

**Table 4. Collinearity statistics (VIF) - Inner model**

|  | VIF |
| --- | --- |
| ATT → GPI | 1.283 |
| GW → GPI | 1.287 |
| PBC → GPI | 1.266 |
| PCE → GPI | 1.353 |
| SN → GPI | 1.327 |
| GW * ATT → GPI | 1.267 |
| GW *SN → GPI | 1.200 |
| GW * PBC → GPI | 1.310 |
| GW * PCE → GPI | 1.209 |

*Source: Author’s compilation, 2024*

Falk & Miller (1992) recommend $R^{2}$ that endogenous variables be ≥ 0.10. An $R^{2}$ ≥ 0.10 ensures that the variance explained by the endogenous variables has practical, as well as statistical, significance. In this study, the value of the R-square which is 0.498 is satisfactory.

**Table 5. Path Coefficients and Hypotheses Testing**

| **Hypothesis** | **Relationships** | **Beta** | **Mean** | **SD** | **t value** | **p-value** | **Decision** |
| --- | --- | --- | --- | --- | --- | --- | --- |
| **Direct effect** | | | | | | | |
| H1 | ATT → GPI | 0.122 | 0.127 | 0.039 | 3.141 | 0.002 | Supported |
| H2 | SN → GPI | 0.234 | 0.236 | 0.040 | 5.812 | 0.000 | Supported |
| H3 | PBC → GPI | 0.262 | 0.259 | 0.043 | 6.156 | 0.000 | Supported |
| H4 | PCE → GPI | 0.232 | 0.234 | 0.040 | 5.863 | 0.000 | Supported |
| **Moderator effect** | | | | | | | |
| H5 | GP*ATT → GPI | -0.105 | -0.103 | 0.033 | 3.185 | 0.001 | Supported |
| H6 | GP*SN → GPI | 0.056 | 0.059 | 0.038 | 1.488 | 0.137 | Not supported |
| H7 | GP*PBC → GPI | 0.053 | 0.051 | 0.041 | 1.282 | 0.200 | Not supported |
| H8 | GP*PCE → GPI | -0.161 | -0.157 | 0.033 | 4.848 | 0.000 | Supported |

*Source: Author’s research, 2024*

Table 5 demonstrates that the results of the structural model and the standardized path coefficient indicated positive effects among the constructs in the structural model.

The results show that attitude has a positive effect on consumers’ green purchase intention (H1: b= 0.122, t= 3.141, p = 0.002) specifying that H1 was supported. This underscores the role of positive perceptions of sustainable fashion in shaping Vietnamese Gen Z consumers' intentions, aligning with previous research (Nguyen et al., 2019; Ahmed et al., 2021). Gen Z’s recognition of sustainable fashion as environmentally beneficial and as a representation of their self-image enhances their likelihood of engaging in green purchases. Subjective norms significantly affect Gen Z consumers’ green purchase intention (H2: b= 0.234, t= 5.810, p =0.000), supporting H2. This suggests that societal trends and peer encouragement motivate Gen Z consumers in Vietnam to adopt sustainable purchasing habits, aligning with prior studies (Rausch & Kopplin, 2022; Salmivaara et al., 2021). Perceived behavioral control positively affects young consumers’ green purchase intention (H3: b b= 0.262, t= 6.156, p= 0.000). Therefore, H3 is supported. This indicates that confidence and access to sustainable fashion products enhance Gen Z consumers' likelihood of engaging in green consumption, consistent with the results of Juschten et al. (2019) and Yadav & Pathak (2016). Finally, perceived consumer effectiveness positively affects young consumers’ green purchase intention (H4: b= 0.232, t=5.863, p=0.000). The findings suggests that Gen Z consumers are more inclined to make green purchases when they believe their actions contribute to environmental sustainability, reinforcing prior studies by Jaiswal & Kant (2018) and Kamalanon et al. (2022).

The effective size can be used to estimate the power of the moderating effect (Henseler & Fassott 2009; Cohen 1990). Based on the $f^{2}$ value, the effect size of the omitted construct for a particular endogenous construct can be determined such that 0.02, 0.15, and 0.35 represent small, medium, and large effects, respectively (Cohen, 1988).

**Table 6. Strength of moderating effects based on the rule of Cohen 1998.**

| Endogenous latent variable | $R^{2}$ | Interaction terms | $f^{2}$ | Effect size |
| --- | --- | --- | --- | --- |
| Green purchase intention | 0.498 | GW* ATT | 0.021 | Small |
|  |  | GW* SN | 0.005 | No effect |
|  |  | GW*PBC | 0.006 | No effect |
|  |  | GW*PCE | 0.051 | Small |

*Source: Author’s compilation, 2024*

The power of the moderating effect is shown in the table 6. According to [Chin et al. (2003](https://www.researchgate.net/publication/220079717_A_Partial_Least_Squares_Latent_Variable_Modeling_Approach_for_Measuring_Interaction_Effects_Results_from_a_Monte_Carlo_Simulation_Study_and_an_Electronic-Mail_EmotionAdoption_Study)), a small effect size does not always imply that the causal preservative effect is insignificant. Chin et al. (2003) stated that even a small interaction effect could make sense under exceptional moderating settings; if the resulting beta changes are expressive, at that time, it is important to consider these conditions. This recommended that the moderating effects of greenwash perception in attitude, and perceived consumer effectiveness on the green purchase intention of Gen Z consumers could be significant.

Table 7 below demonstrates the value of $Q^{2}$. According to Hair et al., (2019), the level of $Q^{2}$ corresponds to the model's predictive ability as follows: 0 < $Q^{2}$ < 0.25: low level of authentication accuracy; 0.25 ≤ $Q^{2}$ ≤ 0.5: Average forecast report accuracy; $Q^{2}$ > 0.5: High forecast accuracy.

The value of $Q^{2}$ in the table indicates the medium predictive relevance.

**Table 7. LV prediction summary**

|  | $Q^{2}$predict | RMSE | MAE |
| --- | --- | --- | --- |
| GPI | 0.47 | 0.731 | 0.575 |

*Source: Author’s compilation, 2024*

***4.2. Moderating Effects Testing***

To model the moderating effects of latent variables in structural equation models, Kenny & Judd (1984) proposed building product terms between the indicators of the latent independent variable and the indicators of the latent moderator variable. These product terms serve as indicators of the interaction term in the structural model. In the present research, the product indicator technique is used to identify and evaluate the moderating power of the effect of greenwash perception on the association (ATT-GPI, SN-GPI, PBC-GPI, PCE-GPI).

Table 5 and Figure 5 suggest that the ATT*GP interaction terms (b =- 0.105, t = 3.185, p = 0.001) and the PCE*GP interaction term (b= -0.161, t= 4.848, p= 0.000) were significant (See Figure 5). Therefore, H5 and H8 have received full support. This suggests that when consumers perceive high levels of greenwashing, their positive attitudes toward sustainable fashion and belief in personal efficacy are diminished, leading to lower purchase intention. These findings align with Rausch & Kopplin (2021) and Rejikumar (2016), emphasizing the significant impact of greenwash perception as a barrier to green consumption. However, the SN*GP interaction terms (b=0.056, t= 1.488, p= 0.137) and the PBC*GP interaction term (b=-0,053, t=1.282, p=0.200) were insignificant. Thus, H6 and H7 have not received support, showing that social influences and perceived control over green purchases remain influential despite skepticism toward corporate sustainability claims.

The slope for the association between attitude, perceived consumer effectiveness, and consumers’ green purchase intention moderated by perceived greenwash was given in Figures 6 and 7 respectively. (See Figure 6,7).

6. The conclusion, implications and limitations are relevance with the study. However the implications should based on the observed variables to suggest more details.

**Authors’ response:** Thank you for your valuable feedback. The implications section has been revised to provide more detailed recommendations based on the observed variables. We have ensured that the implications are closely tied to the study’s key findings. This includes practical examples of how the observed variables can inform strategies to address the identified issues or opportunities.

**Change in manuscript:**

**5. Conclusions and Implications**

***5.2 Theoretical implications***

The research has developed a model to study the factors influencing green purchase intention, with the addition of the moderating effect of greenwashing perception on these relationships with a focus on Generation Z in Vietnam's fashion sector. This is a new and different finding from previous research as the moderating role of greenwash perception has introduced a new dimension to the TPB framework, suggesting that external factors can influence the relationship between attitudes, perceived consumer effectiveness, and sustainable clothing purchase intention. Our results align with Rausch and Kopplin's (2021) argument that greenwash concerns can impact consumers' purchasing decisions. However, we extend their work by demonstrating that greenwash perception, rather than just greenwash concern, plays a crucial moderating role. The study demonstrates how greenwash perception can alter the impact of other factors on green buy intention, providing a comprehensive view of the psychological mechanisms behind sustainable purchasing behavior.

Furthermore, there remains a limited number of studies investigating the moderating effect of greenwash perception on various relationships. Our research has contributed to this field by focusing on a narrower scope, specifically targeting Generation Z individuals in Vietnam within the fashion industry. By doing so, we have provided unique insights into a demographic and market that have been underexplored in existing literature.

***5.3. Managerial implications***

The implications drawn from the research have significant practical relevance for various stakeholders in the context of Greenwashing among Generation Z in Vietnam's fashion industry.

For marketers and organizations, the study emphasizes the necessity of designing marketing strategies that align with Gen Z’s values and concerns. As Gen Z prioritizes sustainable and eco-friendly clothing, businesses may capitalize on this demographic's desire to make a good effect by promoting the environmental benefits of their products and services. Businesses should make investments in programs that advance their environmental awareness and show a sincere dedication to sustainability. This involves training personnel, establishing sustainable practices, and clearly explaining green initiatives. Furthermore, the findings underscored the value of openness and authenticity in green marketing. By transparently communicating the actual environmental impact of their products, businesses can strengthen consumers' belief in the positive effects of their green purchases. Customers from Generation Z penalize companies that are perceived to be inauthentic, underlining the importance of transparent communication regarding actual sustainability measures. This includes precise information on materials, manufacturing methods, and environmental impacts, which may be accompanied by relevant certifications.

The findings can also direct policymakers in encouraging sustainable consumerism and giving youth the tools they need to make wise decisions. Policymakers should impose stronger restrictions and punishments for misleading statements to level the playing field for companies and guarantee that customers have access to correct information. By enacting laws that address problems like resource usage, waste management, and labor standards, policymakers may also aid in the growth of a more sustainable fashion sector. By creating a supportive environment for sustainable businesses, policymakers can encourage innovation and drive positive change.

7. Please check carefully the formatting and typos.

**Authors’ response:** Thank you for your comment. We have thoroughly reviewed the manuscript to ensure proper formatting and correct any typographical errors. This includes a detailed check of headings, citations, references, and the overall layout to maintain consistency and clarity throughout the document.

**Reviewer #4: Based on the research content, I would like to provide the following feedback to the author:**

- The study has chosen a topic that aligns with the current trend of sustainable development. Investigating the impact of "greenwashing" on Gen Z's green purchasing intention is a novel and highly practical direction, especially in the context of the Vietnamese fashion industry, which is undergoing a transition to a sustainable business model.

- The study used appropriate methodologies and conducted a rigorous data analysis. The use of PLS-SEM and simple slope analysis has enabled the author to accurately and reliably test the research hypotheses.

- The research findings provide useful information for fashion businesses in developing effective business and contribute to raising consumer awareness of "greenwashing".

Some suggestions for this study:

- The context and rationale for choosing Vietnam as the research setting need to be clarified. The author should provide more information about the sustainable fashion market in Vietnam, the challenges and opportunities for businesses and consumers.

- The author should discuss the limitations of the study in more depth and propose more specific directions for future research.

Overall, this is a promising study that has the potential to contribute to the development of consumer behavior research. After being revised and supplemented based on the above suggestions, the paper will have higher scientific and practical value.

**Author’s response to the reviewer’s suggestion:**

- The context and rationale for choosing Vietnam as the research setting need to be clarified. The author should provide more information about the sustainable fashion market in Vietnam, the challenges and opportunities for businesses and consumers.

**Authors’ response:** Thank you for your valuable feedback. For the first suggestion about the reason for choosing Vietnam, we have expanded the introduction to provide a clearer context and rationale for selecting Vietnam as the research setting. This includes a discussion of Vietnam's growing sustainable fashion market, and its relevance to the study. Additional details about the challenges for businesses and consumers in Vietnam have been included to highlight the significance of this research.

**Changes in manuscript:**

**1. Introduction**

In Vietnam, the government has implemented the National Strategy for Green Growth for 2021–2030 with a vision for 2050. The strategy includes initiatives to encourage sustainable consumption and promote greener lifestyles. Alongside rapid economic growth and a booming fashion sector (Nayak, 2021), Vietnamese consumers are increasingly aware of sustainability. However, the sustainable fashion industry in Vietnam faces significant challenges, including high production costs, limited resources (Zhang et al., 2018; Jackman & Moore, 2021), and deceptive practices like greenwashing, where companies create false impressions of environmental friendliness (Nguyen et al., 2019). Additionally, Vietnam still lacks comprehensive regulations and enforcement mechanisms to address these issues effectively. To be more precise, greenwashing refers to creating a false impression or presenting misleading information about a company's products being environmentally friendly. It arises from combining poor environmental performance with positive communication about sustainability (Nguyen et al., 2019). While greenwashing has been widely studied, there is a notable lack of research on its prevalence in the fashion industry within developing Asian countries (Yang et al., 2020). This is crucial, as consumers are often willing to pay more for products from companies genuinely committed to sustainability (Chen et al., 2018).

- The author should discuss the limitations of the study in more depth and propose more specific directions for future research.

**Authors’ response:** Thank you for your suggestion. The limitations section has been expanded to include a more in-depth discussion of the study's constraints, such as sample size, geographic focus, and reliance on self-reported data, as well as proposing more specific directions for future research.

**Changes in manuscript:**

**6. Limitations and future reseach direction**

Although this study provides important new information about how Vietnam's Generation Z is affected by their perception of greenwashing, it also highlights its shortcomings and makes recommendations for future research areas. Firstly, the sample size may affect the generalizability of the findingsas it does not fully capture the Vietnam’s Generation Z population. Future research could broaden the scope of studies to include a more representative range of individuals from broader demographic and geographic segments for more comprehensive results. Secondly, the study is limited to the fashion industry, which does not comprehensively reflect the influence of greenwashing across other sectors. Future research could broaden its scope to encompass diverse sectors, such as electronics, food, energy, and beyond, across multiple countries. Finally, the conceptual model used a limited number of antecedents affecting green purchase intention. Future research could incorporate elements like perceived risk, green skepticism, and brand trust while examining mediating and moderating effects of consumer values and cultural influences.
